# Baseline procalcitonin as a predictor of bacterial infection and clinical outcomes in COVID-19: *A case-control study*

**DOI:** 10.1371/journal.pone.0262342

**Published:** 2022-01-13

**Authors:** Natalie J. Atallah, Hailey M. Warren, Matthew B. Roberts, Ramy H. Elshaboury, Monique R. Bidell, Ronak G. Gandhi, Meagan Adamsick, Maryam K. Ibrahim, Rupali Sood, Savo Bou Zein Eddine, Matthew J. Cobler-Lichter, Natalie J. Alexander, Kyle D. Timmer, Christine J. Atallah, Adam L. Viens, Vahe S. Panossian, Allison K. Scherer, Teddie Proctor, Sherrie Smartt, Alyssa R. Letourneau, Molly L. Paras, Sascha Johannes, Jan Wiemer, Michael K. Mansour

**Affiliations:** 1 Division of Infectious Diseases, Massachusetts General Hospital, Boston, MA, United States of America; 2 Harvard Medical School, Boston, MA, United States of America; 3 Department of Medicine, Massachusetts General Hospital, Boston, MA, United States of America; 4 Department of Pharmacy, Massachusetts General Hospital, Boston, MA, United States of America; 5 Department of Surgery, Massachusetts General Hospital, Boston, MA, United States of America; 6 Division of Cardiology, Massachusetts General Hospital, Boston, MA, United States of America; 7 Faculty of Medicine, University of Balamand, Beirut, Lebanon; 8 Faculty of Medicine, American University of Beirut Medical Center, Beirut, Lebanon; 9 Fisher Diagnostics, Part of Thermo Fisher Scientific, Middletown, VA, United States of America; 10 B·R·A·H·M·S GmbH, Part of Thermo Fisher Scientific, Hennigsdorf, Germany; University of South Carolina, UNITED STATES

## Abstract

**Purpose:**

Coronavirus disease-2019 (COVID-19) is associated with a wide spectrum of clinical symptoms including acute respiratory failure. Biomarkers that can predict outcomes in patients with COVID-19 can assist with patient management. The aim of this study is to evaluate whether procalcitonin (PCT) can predict clinical outcome and bacterial superinfection in patients infected with severe acute respiratory syndrome coronavirus-2 (SARS-CoV-2).

**Methods:**

Adult patients diagnosed with SARS-CoV-2 by nasopharyngeal PCR who were admitted to a tertiary care center in Boston, MA with SARS-CoV-2 infection between March 17 and April 30, 2020 with a baseline PCT value were studied. Patients who were presumed positive for SARS-CoV-2, who lacked PCT levels, or who had a positive urinalysis with negative cultures were excluded. Demographics, clinical and laboratory data were extracted from the electronic medical records.

**Results:**

324 patient charts were reviewed and grouped by clinical and microbiologic outcomes by day 28. Baseline PCT levels were significantly higher for patients who were treated for true bacteremia (p = 0.0005) and bacterial pneumonia (p = 0.00077) compared with the non-bacterial infection group. Baseline PCT positively correlated with the NIAID ordinal scale and survival over time. When compared to other inflammatory biomarkers, PCT showed superiority in predicting bacteremia.

**Conclusions:**

Baseline PCT levels are associated with outcome and bacterial superinfection in patients hospitalized with SARS-CoV-2.

## Introduction

Coronavirus disease-2019 (COVID-19) was declared a global pandemic by the World Health Organization (WHO) in 2020. It was first reported in Wuhan, China, and SARS-CoV-2, a single stranded RNA virus, was identified as the causative pathogen [1–4]. To date, the United States has had the highest case numbers rates reaching 28 million total cases with the death toll of 514,000 patients resulting in a case fatality rate of 1.8% with over 114 million cases of SARS-CoV-2 infection worldwide [5].

Mild symptoms of COVID-19 infection are characterized by fever, dry cough, fatigue, and myalgia, and for some, bacterial superinfection may develop. In more severe cases, symptoms progress to acute respiratory distress syndrome (ARDS) and respiratory failure requiring ICU-level care [1–4]. The hallmark of advanced disease is a severe immune dysfunction leading to multiorgan dysfunction [6]. Several risk factors for severe COVID-19 have been identified, including obesity, prior lung disease, cardiac disease, diabetes, and advanced age. However, it remains difficult to predict which patients will develop bacterial superinfection and who will have poor outcomes. Due to the severity of COVID-19, a lack of initial treatment options and concerns for untreated concurrent bacterial infection, there has been widespread use of antimicrobials in the management of COVID-19 patients [7–11], potentially driving antimicrobial resistance, higher rates of *Clostridioides difficile* colitis and medication side-effects.

Procalcitonin (PCT) is a 116 amino acid glycoprotein produced by neuroendocrine thyroid parafollicular C cells and normally below plasma levels of 0.1 ng/mL in healthy individuals [12]. PCT is highly upregulated in nearly all tissues in response to inflammatory cytokines, such as TNF-α, interleukin (IL)-1, and IL-6, commonly triggered by bacterial infection [13]. PCT has been approved by the US Food and Drug Administration (FDA) for prognostication in sepsis as well as antimicrobial de-escalation in lower respiratory tract infection) [14]. As such, PCT is known to be helpful in distinguishing between bacterial and non-bacterial causes of lower respiratory tract infections, with exceptions including severe COVID-19 [1,2,7,15–39], and is particularly useful in pneumonia for antibiotic de-escalation [13,33].

In the setting of the inflammatory response due to SARS-CoV-2 infection, PCT and other inflammatory biomarkers continue to be used as prognostic indicators. Baseline PCT levels measured in patients with SARS-CoV-2 infection that show elevation may be associated with worse COVID-19 disease severity and patient outcomes [1,2,7,15–39]. Confounding correlations between PCT levels and clinical outcomes are observations that possible immune dysregulation triggered by SARS-CoV-2 [40] may result in elevated inflammatory biomarkers, including PCT.

The aim of this study is to evaluate whether PCT concentrations can independently stratify and prognosticate secondary bacterial superinfection and patient outcome in the setting of SARS-CoV-2 infection. We performed a case control study at a tertiary medical center comparing clinical metrics in cohorts of COVID-19 patients, with and without evidence of bacterial superinfection.

## Materials and methods

### Study design and study population

A retrospective observational study of patients who were admitted to a tertiary hospital in Boston, Massachusetts, USA, between March 17 and April 30, 2020, with documented SARS-CoV-2 infection was conducted. This study was approved by the Mass General Brigham (MGB) Institutional Review Board (IRB) in accordance with the Declaration of Helsinki for ethical principles in medical research. Informed consent was waived for this retrospective study as decided by the MGB IRB ethics committee. The data was not anonymized before the study team accessed the medical information.

The study population included admitted adult inpatients (≥ 18 years of age) who had a positive SARS-CoV-2 RT-PCR assay with at least one PCT value. The date but not the exact time of day was noted for PCT collection. The PCT turnaround time was 2–4 hours and performed at the hospital using an Elecsys BRAHMS PCT on a Roche Cobas platform. The resulting PCT value (normal reference range 0.0–0.08 ng/mL) was reported into the medical record with high value cutoff at 0.1 ng/mL without any further guidance provided. Patients were excluded with a presumed, but not confirmed, SARS-CoV-2 diagnosis, or if no PCT determinations were made. Given confounding of PCT values from other common extra-pulmonary infections, patients with negative bacterial cultures but positive urinalysis were excluded.

The electronic medical record system was queried for all positive blood and/or respiratory bacterial cultures and data collected. Positive cultures for organisms commonly classified as a contaminant such as coagulase-negative *Staphylococcus* were reviewed by an adjudication committee to stratify as appropriate.

Demographics, clinical and laboratory data were extracted from the electronic medical records. The ordinal scale, an eight-category scale, was used daily to assess the patient’s clinical status [46]. The categories are ranked as the following: 1 (not hospitalized and no limitation of activities), 2 (not hospitalized, with limitation of activities), 3 (hospitalized, not requiring supplemental oxygen and no longer requiring ongoing medical care), 4 (hospitalized, not requiring supplemental oxygen but requiring ongoing medical care), 5 (hospitalized, requiring supplemental oxygen), 6 (hospitalized, requiring noninvasive ventilation or use of high flow oxygen devices), 7 (hospitalized, receiving invasive mechanical ventilation or extracorporeal membrane oxygenation (ECMO)), and 8 (death) [46].

The collected variables were recorded into a REDCap database [41,42]. In the case of discrepancies, a final decision was adjudicated by the research team. Finally, independent data checks for all collected metrics were performed by the team prior to analysis.

### Statistical analysis

The full study analysis population and its sub-strata were characterized by medians and inter quartile ranges (IQR, 25%-quartile—75%-quartile) for numerical variables and by counts and within-strata percentages for categorical variables. Patient strata (e.g., survival, culture positivity) were compared by Mann–Whitney U test for numerical variables and χ^2^-test for categorical variables (reported p-values without correction for multiple testing). Data distributions were visualized by boxplots with superimposed numerical values with random scatter along the categorical axis of patient strata, and by stacked histograms. Diagnostic and prognostic performance of binary discretized biomarkers (after the application of standard cut-offs) was characterized by sensitivity, specificity, positive predictive value (PPV) and negative predictive value (NPV); estimates were reported with 95% confidence intervals (Clopper and Pearson). Diagnostic and prognostic performance of numerical biomarkers (before cutoff application) was described by receiver operating characteristic (ROC) curves and corresponding area und the curve (AUC) summary measures with 95% confidence intervals [43]. Survival curves of patient strata were estimated according to Kaplan-Meier, plotted with 95% confidence intervals and compared with log-rank test [44]. The added value of PCT in addition to clinical covariates was analyzed with logistic regression (p-values according to Wald test). Statistical analyzes were conducted with R 3.5.1 (checkpoint 3.5.3 for package handling, tidyverse 1.3.0 collection of packages, additional packages rms 5.1–4, ROCR 1.0–7, pROC 1.15.3, survminer 0.4.6 and MASS 7.3–51.4) [45,46].

## Results

**Fig 1:**
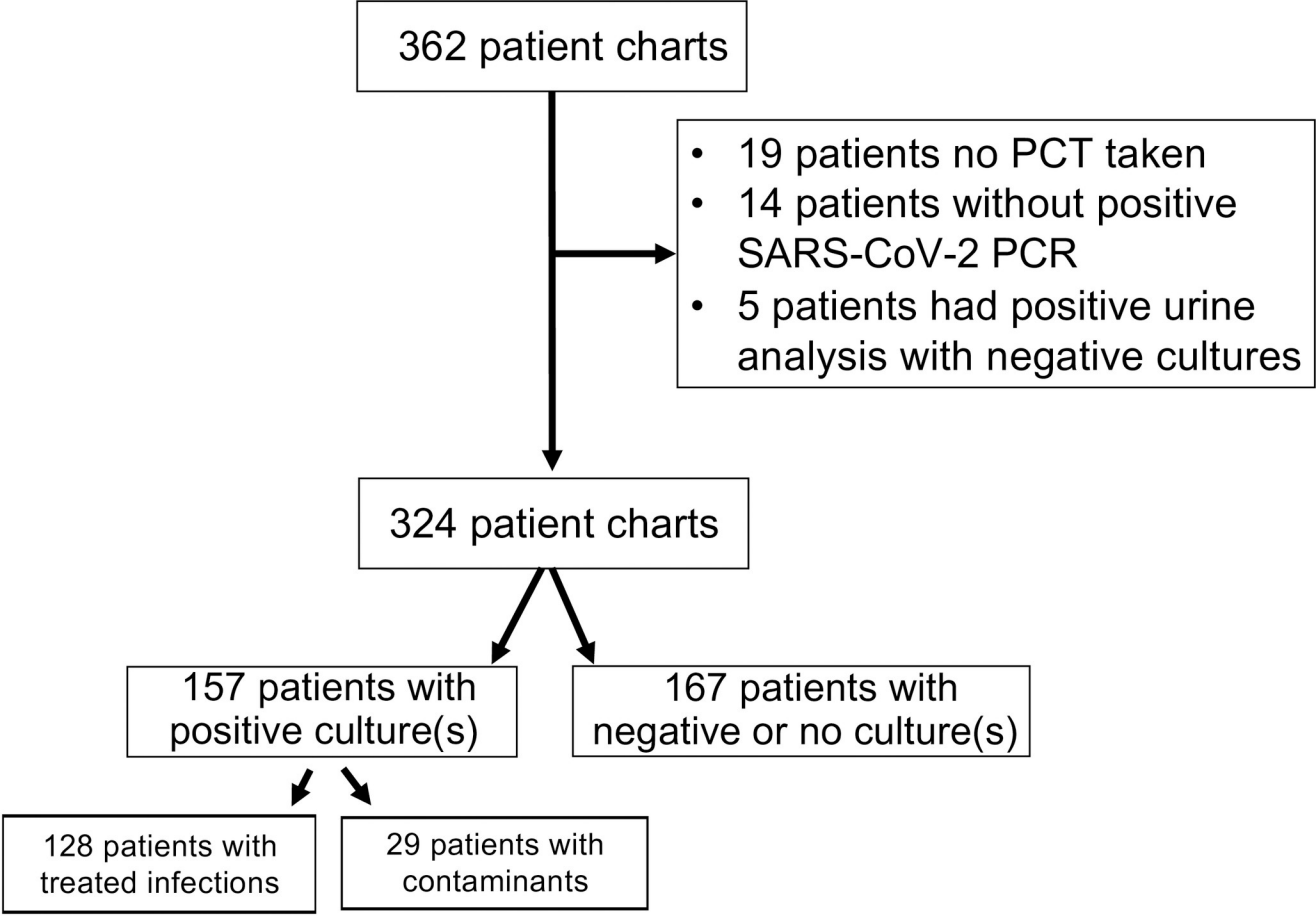
Consort flow diagram of reviewed charts. Information on microbiology refers to cultures taken at any point during hospitalization.

A total of 362 patient charts were reviewed, of which a total of 38 charts were excluded with a final of 324 charts included in the analysis (**Fig 1**). A total of 157 patients had positive blood and/or respiratory cultures, and 167 patients had either negative or no bacterial cultures. Out of the 128 patients treated for true infections, 26 had at least one positive blood culture, 120 had at least one positive sputum culture, and 18 had at least one positive blood and sputum culture. Overall, 303 patients (94% of the 324 total charts) had a baseline PCT level, and the remaining 21 patients (6% of total charts) had a PCT level taken during their hospitalization but not on admission. A smaller subset of patients (n = 144; 44%) had multiple PCT measured. Radiology imaging was assessed with 94.1% of all subjects having a radiographic finding. Baseline patient characteristics are outlined (**Table 1**).

**Table 1:** Baseline patient characteristics.

	Total	Survived Until day 28	Deceased by day 28	P-Value
**Age [years], median (IQR)**	61.5 (50–73.25)	59 (46.25–70)	74.5 (65.25–84.75)	1.20E-10
**Male gender, n (%)**	194 (59.9%)	157 (59%)	37 (63.8%)	0.61
**BMI n (%)**				0.77
< 18.5	2 (0.6%)	2 (0.8%)	0 (0%)	
18.5-< 25	58 (17.9%)	46 (17.3%)	12 (20.7%)	
25-< 30	120 (37%)	100 (37.6%)	20 (34.5%)	
>30	143 (44.1%)	117 (44%)	26 (44.8%)	
**Ethnicity n (%)**				0.00012
Hispanic	129 (39.8%)	119 (44.7%)	10 (17.2%)	
Non-Hispanic	153 (47.2%)	112 (42.1%)	41 (70.7%)	
Unknown	42 (13%)	35 (13.2%)	7 (12.1%)	
**Diabetes n (%)**	128 (39.5%)	99 (37.2%)	29 (50%)	0.18
**Heart Failure n (%)**	45 (13.9%)	26 (9.8%)	19 (32.8%)	6.16E-07
**COPD or emphysema n (%)**	40 (12.3%)	23 (8.6%)	17 (29.3%)	0.00012
**Lab values**				
PCT median (IQR)	0.17 (0.10–0.34)	0.15 (0.09–0.25)	0.32 (0.15–1.17)	8.10E-06

Baseline characteristics of the full study population and comparison between survival and deceased patient cohorts.

The median age of all the patients was 62 years (IQR 50–73). Deceased patients were statistically significantly older than survivors. The median age of the patients in the survival group was 59 years (IQR 46–70), whereas the non-survival group was 75 years (IQR 65–85, p = 1.2E-10). Ethnicity was another varying factor, where non-Hispanic patients had a higher mortality rate of 37% (95%-CI: 28% - 46%) as compared to Hispanics whose mortality rate was 8% (95%-CI: 4% - 15%, p = 2E-16). The non-survival group had a higher prevalence of heart failure being 32.8% compared to 9.8% in the survival group (p = 1E-5). Overall, 12.3% of patients had COPD or emphysema with 29.3% being in the non-survival group and 8.6% in the survival group (p = 4E-5). The proportion of diabetes patients was increased in the non-survival group versus the survival group, 50% vs. 37%, but statistically non-significant (p = 0.1). Despite body mass index (BMI) and diabetes mellitus (DM) being known COVID risk factors, no significant difference was observed between the two groups probably due to limitations in sample size and non-numerical coding (BMI reported in 4 categories, **Table 1**).

The most common clinical symptoms were dyspnea (75.3%), cough (70.1%), fever (69.8%), myalgia (34.0%), fatigue (27.8%), diarrhea (25.3%) and nausea/vomiting (22.8%). Baseline inflammatory markers differed between those surviving to day 28 and non-survivors; baseline PCT level in the survival group had a median of 0.15ng/mL (IQR 0.09–0.25), whereas the non-survival group had a median baseline PCT level of 0.32ng/mL (IQR 0.15–1.17, p = 8.1E-06) (**Table 1**).

**Fig 2:**
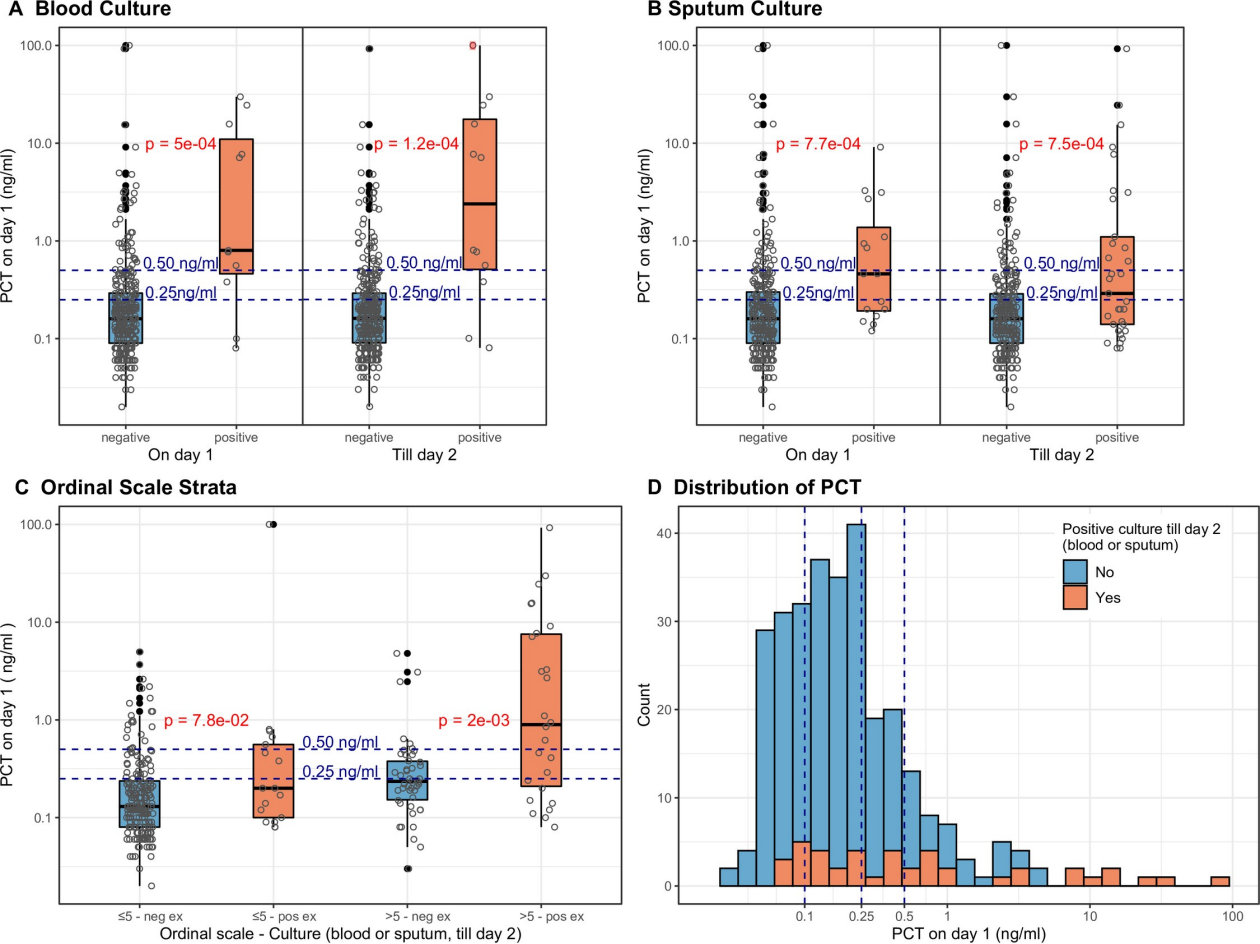
Distribution of baseline PCT in microbiologic strata. Blood (Panel A) and sputum (Panel B) cultures were recorded from day 1 through day 2 of hospitalization. Values shown as open circles with horizontal scatter and summarized by box plots, values beyond whiskers additionally represented by filled circles. Distribution of baseline PCT in ordinal scale and microbiologic strata (Panel C). Stacked histogram of baseline PCT according to microbiology (Panel D).

To further study the role of PCT in the clinical course of patients infected with SARS-CoV-2, the overall distribution of baseline PCT levels was analyzed. The majority of patients who survived to day 28 or had negative cultures had a PCT level of <0.25 ng/mL, and as PCT levels rose, survival decreased (**Fig 2, panel D and S1 Fig**). Similarly, patients who were discharged were more likely to have baseline PCT levels of <0.25ng/mL as compared to patients who were deceased or still hospitalized by day 28.

The association of baseline PCT levels with secondary bacterial superinfection was investigated. Patients were categorized into two groups: the first group included individuals with no bacterial cultures collected, those with negative bacterial cultures and those considered to be contaminants. Whereas the second group was comprised of patients who were treated for true bacteremia and secondary bacterial pneumonia as judged by the treating medical team. Moreover, these two categories were further subdivided to true positive blood cultures and positive respiratory cultures. Baseline PCT levels were significantly higher for the group of patients who were treated for true bacteremia on hospital days 1 (median (IQR): 0.80ng/mL (0.47–11.70), p = 0.0005) and till day 2, meaning the culture was taken on admission day or the day after (13 patients (4%), median (IQR) 3.96ng/mL (0.52–17.90), p = 0.0001) compared with the non-bacterial infection group at the same time points (median (IQR): 0.16ng/mL (0.09–0.29) and 0.16ng/mL (0.09–0.29), respectively) (**Fig 2, Panel A**). Similar differences were observed between patients treated for true bacterial pneumonia as compared with patients who were deemed not to have bacterial pneumonia on hospital days 1 (18 patients (6%); 0.46ng/mL (0.19–1.50) vs. 0.16ng/mL (0.09–0.30), p = 0.0008) and till day 2 (36 patients (11%); 0.29ng/mL (0.14–1.10) vs. 0.16ng/mL (0.09–0.29), p = 0.0008) (**Fig 2, Panel B**). 188 of 260 patients in the negative bacterial cultures group (72.3%, blood or sputum till day 2) had a PCT level of less than 0.25ng/mL (group median (IQR): 0.16ng/mL (0.09–0.27)), whereas 25 of 43 patients in the positive bacterial cultures group (58.1%) had a PCT level larger or equal to 0.25ng/mL (group median (IQR): 0.46ng/mL (0.14–2.91), p = 4.0E-6 for negative vs. positive bacterial cultures group, **Fig 2, Panel D**). Of note, out of the 324 patients, 24 (7.4%) had creatinine greater than 3.0 on admission. All the patients with kidney insufficiency had pct greater than 0.25 ng/mL and 11 (45.8%) of them had a positive blood and/or sputum culture with a similar correlation to the overall cohort analysis.

To better correlate clinical disease severity with PCT in patients with SARS-CoV-2 infections, a daily assessment using the NIAID ordinal scale and the Pneumonia Severity Index (PSI) was performed [47–49]. For hospital day 1 ordinal scale, patients were grouped in either of two groups: ordinal scale > 5 (positive) or ≤5 (negative) (**Fig 2, Panel C**). In the less severe group of patients with ordinal scale ≤5, patients with true positive bacterial cultures (0.20ng/mL (0.10–0.56) had higher PCT levels than the negative cultures group (0.13ng/mL (0.08–0.24) (p = 0.08) (**Fig 2, Panel C**). Similarly, patients in the more severe group, as defined by ordinal scale >5, with positive bacterial cultures had significantly higher PCT levels as compared to the group of patients in the same severity group but without positive cultures (0.90ng/mL (0.21–7.54) vs. 0.24ng/mL (0.15–0.38), p = 0.002) (**Fig 2, Panel C**).

**Fig 3:**
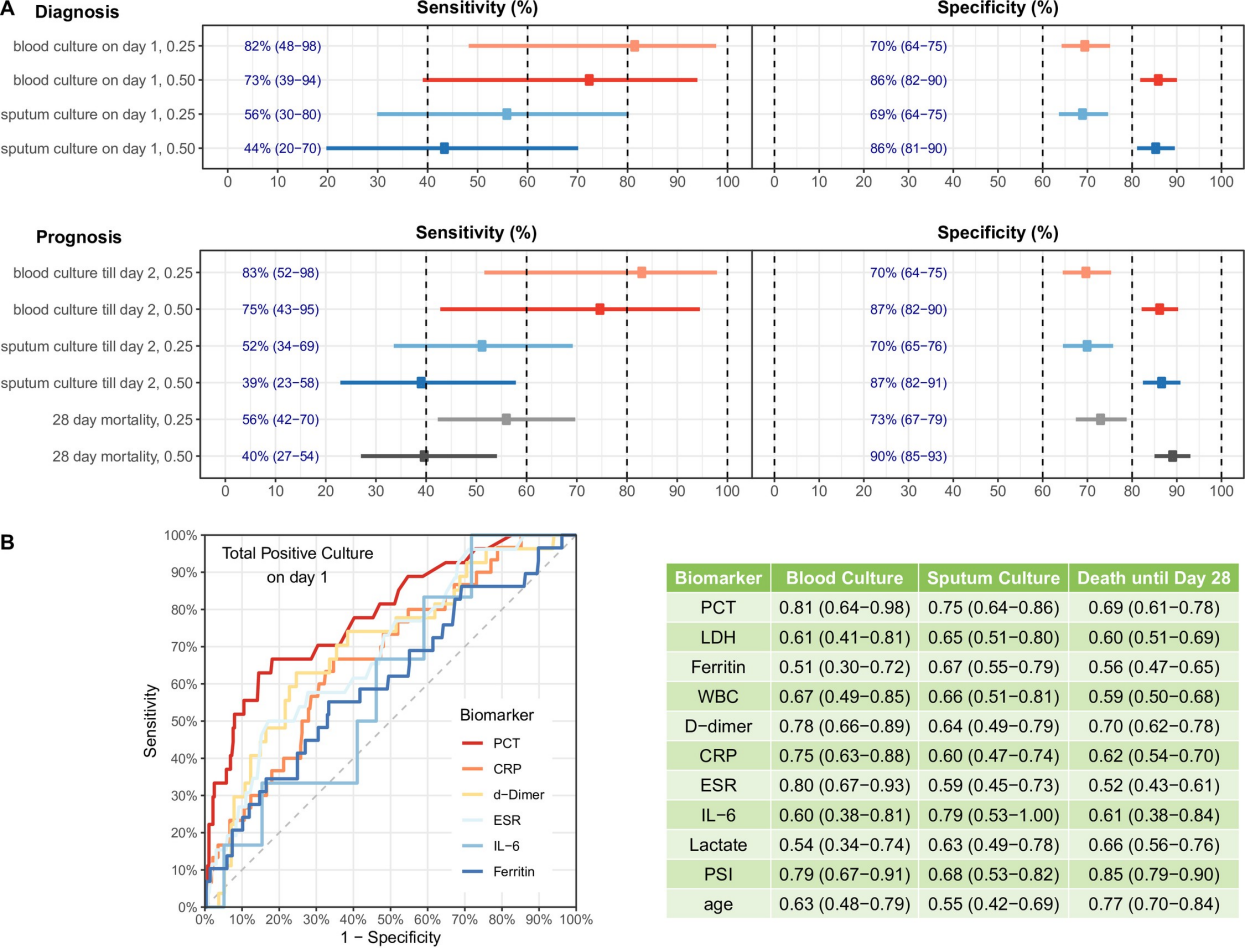
Diagnostic and prognostic performance of PCT levels in determining bacteremia, sputum culture and death by day 28 (Panel A). ROC curves for inflammatory biomarkers (Panel B, left). Biomarker AUCs for determination of death, blood, and sputum cultures (Panel B, right).

When examining both prognostic and diagnostic value, PCT performed better with positive bacterial blood cultures as compared to sputum cultures. For the diagnosis of true bacteremia on hospital day 1, baseline PCT levels had a sensitivity of 82% and specificity of 70% when using the cut-off of 0.25ng/mL. These data contrast with the diagnosis of positive sputum cultures on hospital day 1, where PCT levels had a specificity of 86% when using a cut-off of 0.5ng/mL, but with decreased sensitivity to 44% (**Fig 3, Panel A**). As for the prognostic value of PCT for bacteremia or respiratory infection till hospital day 2 or death by day 28 with a cut-off of 0.25 ng/mL, the sensitivity was 83%, 52%, 56%, respectively, while the specificity was 70%, 70%, 73%, respectively for the cut-off of 0.25 ng/mL. PCT has been shown to predict bacteremia with high NPV and PPV [50–52]. In our COVID-19 cohort, our results show a PCT cut-off of 0.5 ng/mL and 0.25 ng/mL for either bacteremia or bacterial pneumonia performs with a NPV of 95.3% and 95.6% and a PPV of 31.3% and 18.6%, respectively. To address the possibility of bias stemming from the microbiologic collection process (poor specimen collection or missed collection), correlations between PCT and clinical outcomes were performed using a subgroup analysis limited to only those subjects with confirmed, submitted blood or sputum cultures and deemed to represent true infection. Results show similar associations to the general cohort suggesting elevations in PCT do correlate with clinical outcome regardless of submitted microbiologic specimens in this study.

A mixture of gram negative and gram-positive organisms as well as rare fungi were identified in positive blood and sputum cultures. Overall, there were 573 sputum cultures taken; 265 of those were true positives and 308 were negative or contaminants. Some patients had more than one positive culture throughout their hospitalization. Out of 265 positive sputum cultures, the pathogens identified were methicillin-sensitive *Staphylococcus aureus* (MSSA) (n = 106, 40.0%), *Klebsiella* species (n = 54 20.4%), coagulase negative *Staphylococcus* (n = 35, 13.2%), *Pseudomonas aeruginosa* (n = 33, 12.5%), *E*. *coli* (n = 22, 8.3%), MRSA (n = 21, 7.9%), *Streptococcus pneumoniae* (n = 4, 1.5%), and other (n = 57, 21.5%). Yeast/mold (n = 60, 22.6%) were identified as co-cultures in specimens. Out of 35 positive blood cultures, the pathogens identified were coagulase negative *Staphylococcus* (n = 18, 51.4%), MSSA (n = 4, 1.5%), *Klebsiella* species (n = 3, 8.6%), *Pseudomonas aeruginosa* (n = 3, 8.6%), *Streptococcus pneumoniae* (n = 1, 2.9), other (n = 8, 22.9%). There were four positive urine antigen tests of which three were positive for *Streptococcus pneumoniae* and one was positive for *Legionella*.

To clinically correlate PCT with other inflammatory biomarkers, a comparison of AUC was performed. The results indicate that PCT had a highly predictive value for the clinical outcomes such as death, positive bacterial blood and sputum cultures with an AUC of 0.69, 0.81, 0.75 respectively (PCT vs ESR, p = 0.048; PCT vs CRP, p = 0.047; PCT vs ferritin, p = 0.01 for positive cultures on day 1) and was superior in predicting bacteremia as compared to other inflammatory biomarkers (**Fig 3, panel B**). Moreover, PCT levels weakly, but positively correlated with an increasing PSI score with R^2^ = 0.221.

**Fig 4:**
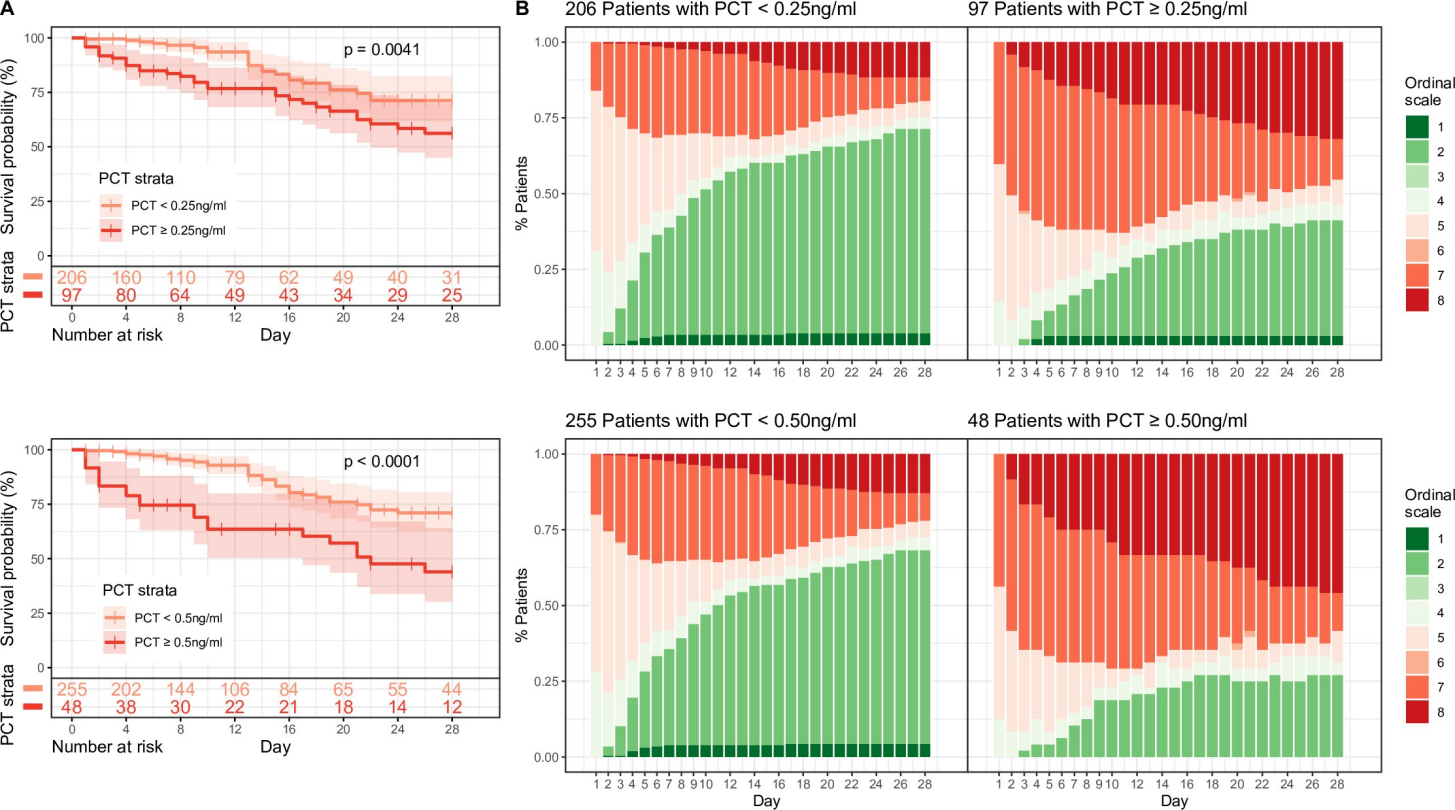
Kaplan-Meier survival plots are separated by PCT strata over time (Panel A). Clinical score (ordinal scale) is plotted over time and stratified by baseline PCT levels (Panel B).

The dependency of the development of the NIAID Ordinal scale on PCT was further analyzed by plotting the data stratified by baseline PCT levels with cut-offs 0.25 ng/mL and 0.5 ng/mL. As PCT cut-off levels increased the percent of patients with more severe ordinal scales proportionally increased as compared to patients with a less severe disease course (**Fig 4, Panel B**)

Next, the association of baseline PCT on clinical metrics including patient survival, time to disposition, duration of antimicrobial therapy was performed. Survival in accordance with the baseline PCT concentration was demonstrated with Kaplan-Meier curves. Hospital day 1 PCT levels were stratified by cut-offs 0.25 or 0.5 ng/mL and survival was plotted over time. A statistically significant lower survival rate was found in above cut-off strata vs. below cut-off strata (**Fig 4, Panel A**).

Hospitalized patient disposition, including discharged, still hospitalized, and deceased by hospital day 28, were correlated with baseline PCT levels. PCT levels were significantly higher in the deceased group and those patients who remained hospitalized compared to those individuals who were discharged (p = 4E-7 and p = 3E-6, respectively) (**S1 Fig**). There was no difference in the baseline PCT levels between patients who were deceased and still hospitalized (p = 0.15). In addition, hospital day 1 PCT levels were significantly different between the group of patients who were admitted to the ICU within their hospital stay versus the group of patients who were treated on medicine wards (p = 3E-08) (**S1 Fig**).

Duration of antibiotic therapy was compared among the four PCT strata, PCT<0.1, ≥0.1–0.25, ≥0.25–0.5 and ≥0.5 ng/mL. As baseline PCT levels increased, the length of antimicrobial therapy expanded in duration (**S2 Fig**). The results were significant between the first two PCT strata (PCT<0.1 versus PCT ≥0.1-.25) (p = 0.00024) (**S2 Fig**). Using time to event plots, multiple correlations were examined, and baseline PCT was shown to be effective in predicting time to discharge, where a significant difference was found between patients who had a PCT value of <0.1 and patients who had a PCT value ≥0.1 and <0.25 (p = 9E-03). In addition, baseline PCT, at higher levels, was able to predict time to positive blood and sputum cultures likely reflecting disease burden at admission (mean discharge day low PCT versus mean discharge day high PCT, p = 0.000098) (**S3 Fig**).

Finally, to further illuminate the association of PCT with clinical outcome prediction and secondary bacterial infection, logistic regressions were performed including the covariates age, BMI<30kg/m^2^, smoking status, history of malignancy, heart failure, COPD or asthma, diabetes, transplant, and a baseline creatinine of >1.2 identified as potential confounders. Complete case analysis was conducted comprising 293 patients. Following corrections, PCT remained a highly significant predictor for secondary bacterial infection, positive blood (OR = 1.62, CI [1.15, 2.28], p = 5.8E-03) and sputum cultures (OR = 1.44, CI [1.10–1.89], p = 8.7E-03), as well as for ventilation (OR = 1.47, CI [1.22–1.77], p = 0.000039), death (OR = 1.39, CI [1.15–1.69], p = 0.00077), and ICU admission (OR = 1.48 CI [1.23–1.78], p = 0.000036) by day 28.

## Discussion

SARS-CoV-2 infections continue to present a global challenge, with multiple surges related to viral variants are expected. As such, biomarkers capable of providing appropriate guidance in the medical management, determination of patient outcome and clinical severity are of the utmost importance. This study examines baseline PCT strata over time and its ability to predict secondary bacterial infection and patient outcome in patients with SARS-CoV-2 infection.

PCT has been useful as a diagnostic indicator to discriminate between bacterial or viral infection, and as a prognostic determinant to assist with antimicrobial de-escalation reflecting that proper source control has been achieved. Our results show that biomarkers such as PCT appear to contribute important information for the prediction of death, ICU admission and secondary bacterial infection, especially if the PCT is negative (NPV >95%). These data have the potential to clinically aid treatment teams in establishing accurate patient prognosis and treatment, although the increase in PCT requires careful consideration and interpretation in the setting of SARS-CoV-2 infection as COVID-19 is known to result in immune dysfunction with a possible increase in inflammatory biomarkers [40]. In our study, baseline PCT levels were positively associated with the daily ordinal scale and PSI, confirming PCT as a predictive biomarker of COVID-19 severity in hospitalized patients. PCT levels correlated with microbial outcomes but given the use of significant amounts of antimicrobials, the possibility that overgrowth of normal flora resulting in skewed microbiologic identification exists [53].

PCT showed a superior role in predicting secondary bacterial blood stream infections compared to other biomarkers. In the correct clinical setting, PCT provides additional granularity to assist in appropriate de-escalation of antibiotics. Patients with a low PCT (<0.25 ng/mL) were unlikely to have positive blood or sputum cultures with a negative predictive value of over 95%. In our cohort, PCT was also associated with length of stay, with high baseline PCT being associated with longer hospitalization. The ability to determine the likely duration of hospitalization at admission can assist in hospital resource utilization.

Other studies have shown that lower PCT levels are associated with lower duration of antibiotic therapy, where the median days of therapy in patients with a PCT >0.25 ng/mL was 5 days versus 2 days in patients with a PCT level <0.25 ng/mL (p<0.001) [20]. A negative PCT group also had a lower mortality rate, ICU rate, less antibiotic usage and hospital length of stay [20,54,55]. While investigations show lower antibiotic usage in patients with a PCT value <0.5 ng/mL [34], other studies demonstrate opposing results with a lack of benefit from a stewardship/PCT strategy [56].

Increased PCT levels >0.5 ng/mL are associated with a higher risk of severe SARS-CoV-2 infection [14,25,26,30,37]. In fact, report of any substantial increase in PCT raises suspicion of bacterial superinfection and/or a more severe SARS-CoV-2 infection clinical course [17]. Correlations of prolonged mechanical ventilation have been made with PCT level >0.1 ng/mL as compared to <0.1 ng/mL [15]. However, unlike our cohort, there was no significant difference in mortality rate or time to intubation between both groups [15].

Throughout the COVID-19 pandemic, studies have suggested an association with PCT and more severe clinical status. We now showed the additional capability of PCT to stratify those patients by several important clinical metrics including worse outcomes, disposition, disease severity scales such as the ordinal scale and critical microbiology allowing healthcare providers to raise reasonable suspicion of bacterial secondary infection.

### Limitations

Several limitations exist including the limited availability of follow-up PCT values for patients hospitalized with SARS-CoV-2. As opposed to other inflammatory biomarkers such as CRP, PCT was ordered less frequently with the majority of our database having only a single baseline PCT value, which raises the possibility of a missed rise in PCT with early infection. Future prospective studies should consider serial PCT measurements to fully address the dynamic PCT changes in the setting of COVID-19 and superinfections.

Inference of patient capacity for providing a sputum (too ill or incapable of producing a sputum culture) presents another limitation of this study since these events were counted as negative cultures. In addition, there are inherent limitations to a sputum analysis, which also raise the possibility that a negative sputum culture result in the setting of elevated PCT values may reflect poor sensitivity and/or accuracy of standard sputum culture as a diagnostic of infection. In fact, some studies have relied on more invasive, albeit more accurate, analysis for the determination of lower respiratory tract infection including bronchoscopy [57], which resulted in a higher sensitivity for the detection of bacterial superinfection. Future prospective trials with more accurate pathogen analysis, such as bronchoscopy or respiratory multiplex PCR diagnostics, would enhance correlations of true invasive infectious states with biomarkers such as PCT.

Additional limitations exist within patient-specific inherent variables that can influence biomarkers including PCT [58]. For examples, chronic renal function has been associated with a rise in PCT independent of infection. In our cohort, we see a positive trend when correlating increasing creatinine with PCT although a rise in PCT remains noted in the setting of true infection.

Finally, this retrospective study is a single site study, which has the potential to introduce both geographic and patient population bias. A prospective, multi-center randomized controlled trial is essential to study the clinical utility of PCT in SARS-CoV-2.

## Conclusion

In conclusion, our study confirms the diagnostic and prognostic value of serum PCT levels in patients with SARS-CoV-2 infection. While elevated PCT levels in COVID-19 patients is a pattern consistent with a bacterial superinfection and associated with increased mortality over 28 days, it needs to be interpreted with caution in the setting of COVID-19-related immune dysfunction. In contrast, a low serum PCT suggests that any detected potential bacterial pathogen is likely colonizing and not invasive. PCT levels were positively associated with patient outcome and secondary bacterial infection. Moreover, PCT correlated with duration of admission, survival time and duration of antimicrobial therapy. These data suggest PCT as a useful prognostic biomarker in the clinical course of hospitalized COVID-19 patients, while low PCT may potentially assist in limiting or de-escalating unnecessary antibiotic treatment. Additional prospective randomized controlled trials are essential to study the clinical impact of PCT in this patient population.

## Supporting information

S1 Fig Boxplots of baseline PCT stratified by clinical outcome (Panel A) and ICU level care (Panel B).
(PDF)

S2 Fig Total days of antimicrobial therapy by PCT strata. (PDF)

S3 Fig Time to event (left: Discharge, center: Bacterial pneumonia, right: Bacteremia) by PCT strata. (PDF)

S1 Raw data (CSV)
