# Supplementary material for: Baseline procalcitonin as a predictor of bacterial infection and clinical outcomes in COVID-19: A case-control study
Source: PLoS One. 2022 Jan 13;17(1):e0262342. doi: 10.1371/journal.pone.0262342 (PMC8758006; doi:10.1371/journal.pone.0262342)
Supplement: S1 Fig — Boxplots of baseline PCT stratified by clinical outcome (Panel A) and ICU level care (Panel B). (PDF) [file pone.0262342.s001.pdf]

# Supplementary Figure 1

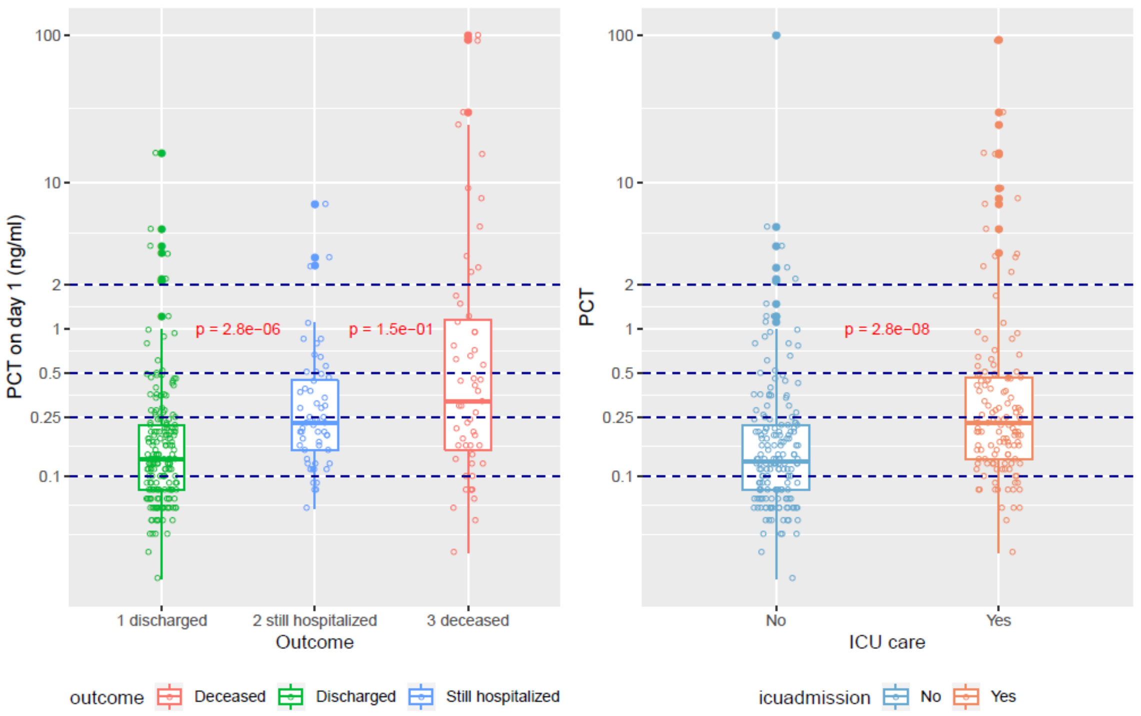


| Comparison | p-value | Assessment |
|---|---|---|
| Discharged vs. Still hospitalized | 2.8E-06 | significant |
| Discharged vs. Deceased | 3.6E-07 | significant |
| Still hospitalized vs. Deceased | 0.15 | not significant |
| ICU care yes vs. no | 2.8E-08 | significant |
