# Supplementary figures and images for: Baseline procalcitonin as a predictor of bacterial infection and clinical outcomes in COVID-19: A case-control study
Source: PLoS One. 2022 Jan 13;17(1):e0262342. doi: 10.1371/journal.pone.0262342 (PMC8758006; doi:10.1371/journal.pone.0262342)

# Supplementary Figure 2

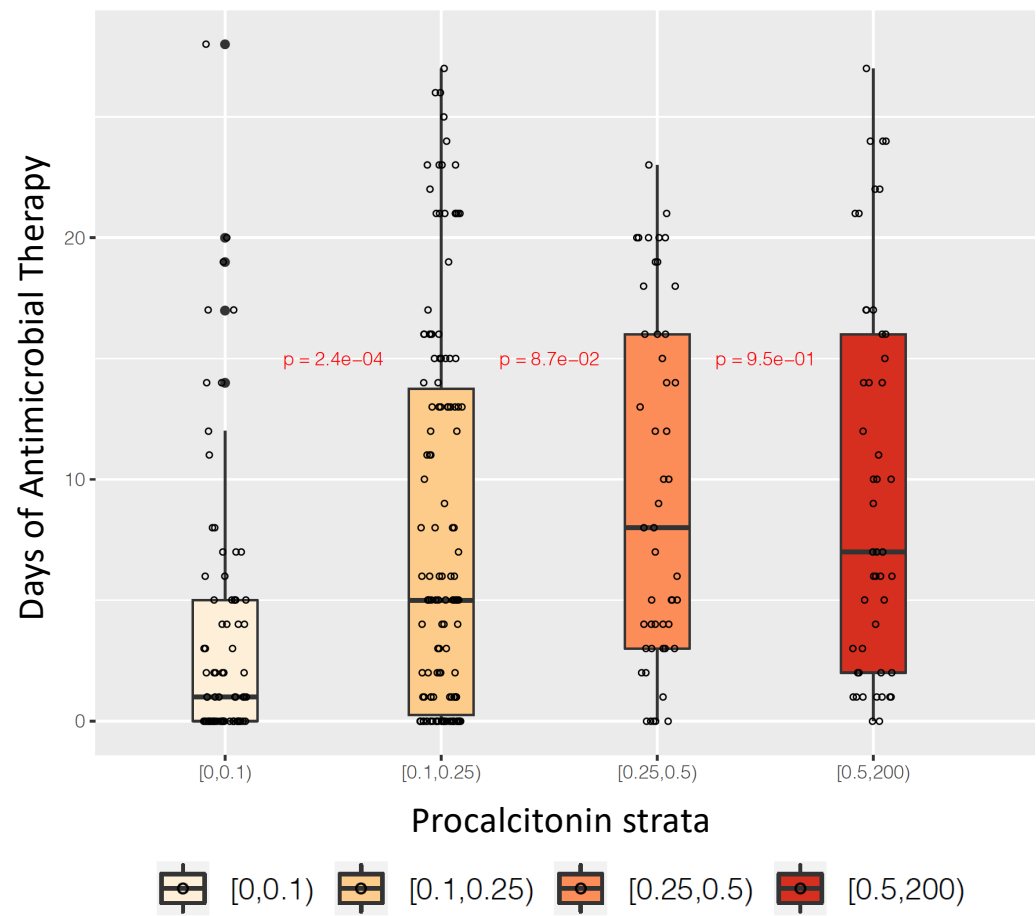

Supplement: S2 Fig — (PDF) [file pone.0262342.s002.pdf]

# Supplementary Figure 3

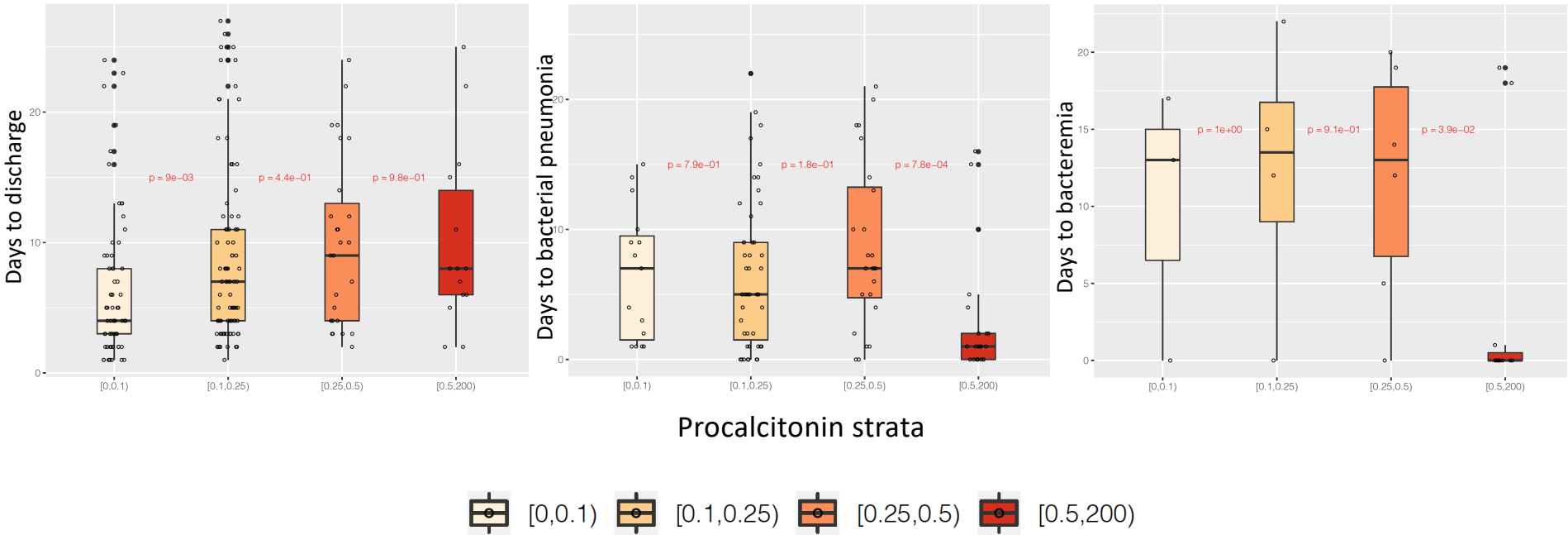

Supplement: S3 Fig — (PDF) [file pone.0262342.s003.pdf]
